# Analyzing Complex Longitudinal Data in Educational Research: A Demonstration With Project English Language and Literacy Acquisition (ELLA) Data Using xxM

**DOI:** 10.3389/fpsyg.2018.00790

**Published:** 2018-06-05

**Authors:** Oi-Man Kwok, Mark Hok-Chio Lai, Fuhui Tong, Rafael Lara-Alecio, Beverly Irby, Myeongsun Yoon, Yu-Chen Yeh

**Affiliations:** ^1^Department of Educational Psychology, Texas A&M University, College Station, TX, United States; ^2^Center for Research & Development in Dual Language & Literacy Acquisition (CRDLLA), College Station, TX, United States; ^3^School of Education, University of Cincinnati, Cincinnati, OH, United States; ^4^Department of Educational Administration and Human Resource Development, Education Leadership Research Center, Texas A&M University, College Station, TX, United States

**Keywords:** longitudinal data analysis, multilevel structural equation models, educational psychology, intervention, bilingual education

## Abstract

When analyzing complex longitudinal data, especially data from different educational settings, researchers generally focus only on the mean part (i.e., the regression coefficients), ignoring the equally important random part (i.e., the random effect variances) of the model. By using Project English Language and Literacy Acquisition (ELLA) data, we demonstrated the importance of taking the complex data structure into account by carefully specifying the random part of the model, showing that not only can it affect the variance estimates, the standard errors, and the tests of significance of the regression coefficients, it also can offer different perspectives of the data, such as information related to the developmental process. We used xxM (Mehta, 2013), which can flexibly estimate different grade-level variances separately and the potential carryover effect from each grade factor to the later time measures. Implications of the findings and limitations of the study are discussed.

## Introduction

**Figure 1:**
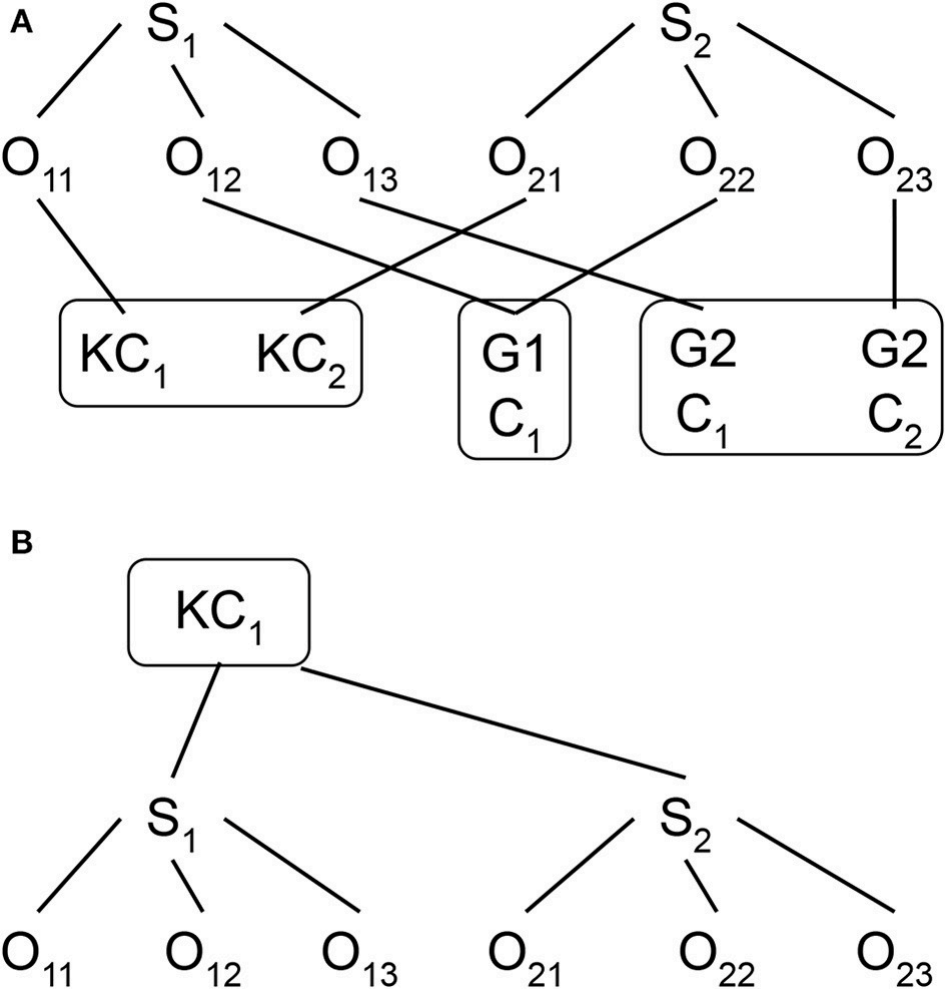
**(A)** Model 2 data structure with repeated measures cross-classified by students and classrooms. O, Observation; S, Student. KC, Kindergarten classroom; G1, Grade 1; C1, Classroom 1; G2, Grade 2; C2, Classroom 2. **(B)** Model 1 data structure with repeated measures nested within students in kindergarten classrooms.

Educational researchers have always involved complex data structure. For example, in cross-sectional studies, students are likely nested within classrooms and schools at a particular time point (i.e., a strictly hierarchical structure), and while they may come from different neighborhoods, neighborhoods and schools are not nested but crossed with each other (i.e., a cross-classified structure). Similarly, for longitudinal data, repeated measures (e.g., reading achievement test scores collected at different grade levels from the same student) are nested within students while the students are likely to change classrooms over the course of study. A change of classroom results in a non-strictly hierarchical, but cross-classified structure, with repeated measures now nested within both students and classrooms, while students and classrooms are crossed with each other (see Figure 1A). Without adequately taking into account all these complex data structures, educational researchers not only may obtain biased parameter estimates and standard errors, but also they miss the opportunity to uncover important phenomena from their data.

Although most educational researchers realize the importance of taking into account the complex data structure when they analyze their data, they may not be aware of how to *fully* address the complex data structure in their analysis and, as a result, they may only *partially* take into account the data structure. For instance, researchers may analyze the cross-classified data structure (e.g., repeated measures nested within students and classrooms, as presented in Figure 1A) by treating it as a strictly hierarchical data structure with the exclusion of the non-kindergarten classroom effect (e.g., first and second grade), as presented in Figure 1B. Without fully addressing the complex data structure, this mis-specified model may lead to a biased estimation of both fixed and random parameters and to incorrect significance tests for the parameter estimates (Meyers and Beretvas, 2006; Luo and Kwok, 2009).

The purpose of this paper was to demonstrate how to analyze this type of complex data structure with the use of data from the Project English Language and Literacy Acquisition (ELLA), a large-scale longitudinal study. The researchers intervened with and followed English language learners (ELLs) from kindergarten to third grade, which was funded by the U.S. Department of Education (Grant Number: R305P030032).

We first provide a brief review of the Project ELLA and the data derived from it. We, then, analyze the data with the commonly used hierarchical linear model [HLM] approach. We subsequently move from this HLM model to the more complex cross-classified random effect model (CCREM) which addresses the complex data structure issue by taking into account the classroom effect. However, the CCREM has its own limitations and is unable to address some of the important features of longitudinal data (which is representative of the dataset from Project ELLA), such as the potential carryover effect (i.e., the effect from the previous grade level on the later time measures). To address this special feature, we used the xxM software (Mehta, 2013; may be downloaded from http://xxm.times.uh.edu/), which could flexibly model the carryover effect during the analysis (the corresponding annotated input syntax and outputs are presented in the appendices). Finally, we discuss the implications of the different results based on different models and re-emphasize the importance of taking the carryover effect into account, followed the limitations of the study and directions for future research.

### Project english language and literacy acquisition (ELLA)

Project ELLA (Lara-Alecio, 2003) was a longitudinal, field-based, large-scale, experimental research project following the same group of native Spanish-speaking, English language learners (ELLs) over time (from kindergarten to third grade) in an urban school district in Southeast Texas. For more than 45% of the students in the district, Spanish was their first language was Spanish. The majority of students qualified for free or reduced-price lunch. All the materials and protocols of Project ELLA were approved by Institutional Review Board (IRB) at Texas A&M University.

Texas state law (Texas Education Code, 1995) has prohibited random selection and assignment to specific instructional delivery models in schools on the basis of individual students; therefore, the research team selected schools where structured English immersion (SEI) and/or transitional bilingual education (TBE) were being implemented within the target school district, and they randomly assigned the selected schools to either a control (typical practice) or an experimental (enhanced practice) setting. Hence, in the overall project, the researchers used an experimental design at the school (classroom) level and a quasi-experimental design with target learning outcomes at the student level.

In the current study, we used a partial data set from the original data. This data set included scores on the English version of the Woodcock Language Proficiency Battery–Picture Vocabulary subtest (EWPV) of 876 students at five time points: Time 1 = beginning of kindergarten (2004), Time 2 = end of kindergarten (2005), Time 3 = end of first grade (2006), Time 4 = end of second grade (2007), and Time 5 = end of third grade (2008).

As shown in Table 1, at Time 1, the study contained 24 schools with 56 classrooms and 876 students (46.00% of females and 53.65% of males) between the ages of 49 and 80 months (*M* = 59.72 and *SD* = 5.08); EWPV data were available for 791 students. At Time 2, it contained 24 schools with 56 classrooms, with EWPV data available for 875 students (45.94% of females and 53.71% of males) between the ages of 61 and 92 months (*M* = 71.72 and *SD* = 5.08). At Time 3, it contained 24 schools with 54 classrooms, with EWPV data available for 643 students (46.19% of females and 53.34% of males) between the ages of 73 and 104 months (*M* = 83.84 and *SD* = 5.01). At Time 4, it contained 21 schools with 53 classrooms, with EWPV data available for 440 students (46.82% of females and 52.50% of males) between the ages of 85 and 112 months (*M* = 95.67 and *SD* = 4.61) had data on EWPV. At Time 5, it contained 21 schools with 60 classrooms, with EWPV data available for 373 students (47.99% of females and 51.21% of males) between the ages of 97 and 124 months (*M* = 107.92 and *SD* = 4.64).

**Table 1:** Descriptive statistics.

	**Time 1 (*N* = 876)**	**Time 2 (*N* = 875)**	**Time 3 (*N* = 643)**	**Time 4 (*N* = 440)**	**Time 5 (*N* = 373)**
**Variables**	***N*** **(%)/*****M(SD)***	***N*** **(%)/*****M(SD)***	***N*** **(%)/*****M(SD)***	***N*** **(%)/*****M(SD)***	***N*** **(%)/*****M(SD)***
**Gender**					
Male	470 (53.65%)	470 (53.71%)	343 (53.34%)	231 (52.50%)	191 (51.21%)
Female	403 (46.00%)	402 (45.94%)	297 (46.19%)	206 (46.82%)	179 (47.99%)
Age (months)	59.72 (5.08)	71.72 (5.08)	83.84 (5.01)	95.67 (4.61)	107.92 (4.64)
**Conditions**					
Control	390 (44.52%)	390 (44.57%)	295 (45.88%)	222 (50.45%)	192 (51.47%)
Treatment	486 (55.48%)	485 (55.43%)	348 (54.12%)	218 (49.55%)	181 (48.53%)

*Time 1, beginning of kindergarten; Time 2, end of kindergarten; Time 3, end of first grade; Time 4, end of second grade; Time 5, end of third grade*.

### Ways to analyze complex longitudinal data in educational research

We present three models, of which the first two are commonly used in educational research; namely, the hierarchical linear model (HLM) and the cross-classified random effect model (CCREM). The third, the xxM-UN1 model, is a more advanced and flexible model, which not only takes into account the complex data structure but also provides new modeling feature that allows researchers to examine such effects as potential carryover in longitudinal analysis. The results from these analytic approaches are compared, and the advantages and disadvantages of each model are discussed.

Even though the analyses have been conducted under both multilevel modeling (MLM; i.e., hierarchical linear modeling, HLM) and structural equation modeling (SEM) frameworks, we prefer using the multilevel modeling framework to present the models for our analyses, given its simplicity for comprehension and the equivalence between the two models (Curran, 2003; Bollen and Curran, 2006). For example, the average trend information in MLM is captured by the corresponding time-related latent factors (i.e., the means and variances of these latent factors) whereas the time-related information (i.e., the time frame of the study) is captured by the factor loadings between the time-related latent factors and the observed variables measured over time under the SEM framework. There are additional benefits of using SEM to analyze longitudinal data, including the availability of model fit indices and modification indices (Preacher et al., 2008; Kwok et al., 2010). Moreover, xxM (Mehta, 2013) provides a flexible framework for modeling complex multilevel and longitudinal data such as the carryover effect detailed later.

## Model 1: the traditional three-level multilevel model

Unlike the cross-sectional multilevel model, there is always an important predictor for longitudinal analysis: time. Researchers are particularly interested in examining the average trend of an outcome variable (in this paper, the Woodcock Language Proficiency Battery–Picture Vocabulary subtest; EWPV) over time. Nevertheless, many longitudinal and developmental phenomena are not linear in nature. In other words, the change of the outcome variable will not happen at a constant rate over time. For example, we may have a simple linear time-predicted model, Math = B0 + B1 Time + e, where Math is the math achievement outcome variable, Time is the time predictor with grade year as the unit, and e as the error. B0 is intercept, B1 (positive and significantly larger than zero) is the regression coefficient, which can be explained as one unit changes in time or one grade year passes, and B1 points change in the math achievement score. More importantly, this model implies the constant improvement in math achievement (with B1 points per grade year regardless of the actual grade year in which the students are located). Hence, fitting a nonlinear model rather than assuming a linear trend is common in analyzing longitudinal data (Kwok et al., 2010).

A relatively, more simple way to capture a nonlinear trend is using a piecewise model (Bryk and Raudenbush, 1992; Sayer and Willett, 1998; Snijders and Bosker, 1999; Duncan et al., 2006; Kwok et al., 2010). By dividing the nonlinear growth trend into different linear segments, one can easily understand the nonlinear trend by applying the same straightforward interpretation based on the simple linear growth rate coefficients. The key part of using the piecewise model is to determine how (many pieces) and where to divide the whole time frame into segments.

For our current demonstration, given the data collection time frame, we determined to use a piecewise model containing two pieces to capture the potential nonlinear trend, with the first piece containing the first two time measures (i.e., beginning and end of kindergarten) and the second piece containing the rest of the three time measures (i.e., end of first grade, end of second grade, and end of third grade). As described previously, we proposed analyzing the data with a piecewise model containing two pieces (a.k.a. a two-piece model). By using the traditional HLM, which assumes a strictly hierarchical structure, we have analyzed our data as a three-level model with repeated measures (level 1) nested within students (level 2) and students further nested within their corresponding kindergarten classrooms (level 3) without considering their mobility (i.e., change of classroom in later time points). The corresponding model equations are presented as follows:

**Level 1 (repeated-measure level)**

(1) $$EWPV_{tij}=\pi_{\text{0}ij}+\pi_{\text{1}ij}\;piece1_{tij}+\pi_{\text{2}ij}\;piece2_{tij}+e_{tij},$$

where EWPV is the target outcome variable for the t-th repeated measure from the i-th student of the j-th ***kindergarten*** classroom, piece1 is the first time piece variable, which captures possible changes in EWPV in kindergarten, and piece2 is the second piece variable, which captures possible changes in EWPV from first to third grade.

We used the following coding scheme:

$$\left[\begin{array}{c} \begin{array}{ccc} \; & piece1 & piece2\\ K-begin & 0 & 0\\ K-end & 1 & 0\\ 1stGrade & 1 & 1\\ 2ndGrade & 1 & 2\\ 3rdGrade & 1 & 3 \end{array} \end{array}\right],$$

with piece1 coded as (0,1,1,1,1) and piece2 coded as (0,0,1,2,3) for the five repeated measures. π_0ij_ is the intercept (or the baseline/predicted EWPV score at the beginning of kindergarten) based on the repeated measures from the i-th student of the j-th kindergarten classroom. Similarly, π_1ij_ is the linear rate of change of the first piece (i.e., from the beginning of kindergarten to the end of kindergarten) while π_2ij_ is the linear rate of change of the second piece (i.e., from the end of first to the end of third grade) from the i-th student of the j-th kindergarten classroom. Given that we had 876 students in the data, and we used the repeated measures from each student to fit the above two-piece model, we should have 876 sets of regression coefficients (i.e., π_0ij_, π_1ij_, & π_2ij_), which can be written into the following equations:

**Level 2 (student level)**

(2) $$\begin{array}{l} \pi_{0ij}=\beta_{0j}+u_{0ij}\\ \pi_{1ij}=\beta_{1j}+u_{1ij}\\ \pi_{2ij}=\beta_{2j}+u_{2ij} \end{array}$$

where β_**0j**_ is the average intercept coefficient across all the students within the j-th kindergarten classroom; β_**1j**_ is the average piece1 regression coefficient across all the students within the j-th kindergarten classroom, and β_**2j**_ is the average piece2 regression coefficient across all the students within the j-th kindergarten classroom.

We further obtained the corresponding average coefficient estimates across all kindergarten classrooms, as presented^1^.

**Level 3 (classroom level)**

(3) $$\begin{array}{l} \beta_{0j}=\gamma_{00}+\gamma_{01}treatment_{j}+v_{0j}\\ \;\beta_{1j}=\gamma_{10}+\gamma_{11}treatment_{j}\\ \;\beta_{2j}=\gamma_{20}+\gamma_{21}treatment_{j} \end{array}$$

where γ_**00**_, γ_**10**_, and γ_**20**_ are the average intercept, piece1 and piece2 coefficients across all kindergarten classrooms assuming a nonsignificant treatment effect.

As stated previously, one of the main purposes of the Project ELLA was to examine the effectiveness of the enhanced practice setting (i.e., the treatment condition) on EWPV. To examine this treatment effect, we included the treatment variable in the level-3 equations, given that the randomization was at the classroom/school level. In other words, students from the same kindergarten classroom received the exact same treatment or control materials. **Treatment**_*j*_ is a dummy-coded variable with treatment condition coded as 1 and control condition coded as 0. Hence, if there is a significant treatment effect at intercept, we expect that γ_**01**_ will not be zero and the intercept for the control condition will be γ_00_ whereas the intercept for the treatment condition will be (γ_00_ + γ_01_). Similarly, if there are significant treatment effects at both piece1 and piece2, we would expect that both γ_11_and γ_21_will not be zero and the average piece1 coefficient will be γ_**10**_ for the control condition and γ_**10**_ + γ_**11**_ for the treatment condition, the same as the average piece2 coefficient with γ_**20**_ for the control condition and γ_**20**_ + γ_**21**_ for the treatment condition.

By substituting Equations (2) and (3) back into equation (1), we can get the following overall ***average*** (or ***mean***) model:

(4) $$\begin{array}{l} ÊWPV_{tij}=\gamma_{00}+\gamma_{01}treatment_{j}+\gamma_{10}\;piece1_{tij}+\\ \;\gamma_{11}treatment_{j}{\;}^{*}\;piece1_{tij}\\ \;+\;\gamma_{20}\;piece2_{tij}\;+\;\gamma_{21}treatment_{j}{\;}^{*}\;piece2_{tij} \end{array}$$

The corresponding random effect variances that capture the variation at different levels are as follows:

V(*e*_*tij*_) = σ^2^ (within-student-level variance with the identity structure assumption)

V(*u*_0*ij*_) = τ_00_ (between-student-level intercept variance)

V(*u*_1*ij*_) = τ_11_ (between-student-level piece1 variance)

V(*u*_2*ij*_) = τ_22_ (between-student-level piece2 variance)

V(*v*_0*ij*_) = θ^2^ (kindergarten classroom-level variance). We used the R package xxM (Mehta, 2013) to analyze our data. (The corresponding output for the model may be found in Appendix A1).

### Results of model 1

As presented in Table 2 in the Model 1 (3-Lv HLM) column, almost all the regression coefficients were significant (with the 95% confidence interval [CI], not including zero) except γ_01_ (i.e., the treatment effect at the beginning of kindergarten). Hence, the overall average piecewise model for the control group (i.e., **treatment**_*j*_ = 0) was:

$$ÊWPV_{tij}\;=\;435.6\;+\;13.75\;piece1_{tij}+\;9.64\;piece2_{tij}$$

whereas the overall average piecewise model for the treatment group (i.e., **treatment**_*j*_ = 1) was:

$$\begin{array}{l} ÊWPV_{tij}=435.6-2.43(1)\;+\;13.75\;piece1_{tij}\;+\;2.41(1)\\ {\;}^{*}piece1_{tij}\;+\;9.64\;piece2_{tij}\;+\;1.60(1){\;}^{*}\;piece2_{tij}, \end{array}$$

which could be further reduced to:

$$ÊWPV_{tij}=433.17+16.16\;piece1_{tij}\;+\;11.24\;piece2_{tij}.$$

**Table 2:** Summary of 3-Level HLM, CCREM, and xxM-UN1 model results.

	**Model 1: 3-Lv HLM**		**Model 2: CCREM**		**Model 3: xxM-UN1**	
**FIXED**						
Intercept (γ_00_)	435.60^*^	[432.91, 438.28]	436.99^*^	[434.31, 439.67]	437.07^*^	[434.16, 440.00]
Piece 1 (γ_10_)	13.75^*^	[11.96, 15.54]	13.15^*^	[11.36, 14.94]	13.12^*^	[11.51, 14.72]
Piece 2 (γ_20_)	9.64^*^	[8.95, 10.34]	9.47^*^	[8.23, 10.71]	9.66^*^	[8.90, 10.44]
Treatment (γ**_01_**)	−2.43	[−7.41, 2.61]	−3.12	[−7.20, 1.03]	−7.06^*^	[−11.96, -1.94]
P1 × Treat (γ**_11_**)	2.41^*^	[0.01, 4.80]	3.42^*^	[1.04, 5.81]	3.46^*^	[1.32, 5.63]
P2 × Treat (γ**_21_**)	1.60^*^	[0.59, 2.62]	0.59	[−1.36, 2.53]	1.42^*^	[0.23, 2.57]
**RANDOM**						
Student
Intercept (τ**_00_**)	137.36		157.04		193.44	
P1 (τ**_11_**)	2.13		3.52		29.37	
Cov(Int, P1)	−17.10		−23.50		−39.80	
P2 (τ**_22_**)	0.08		0.16		3.66	
Cov(Int, P2)	−3.22		−5.02		−13.56	
Cov(P1, P2)	0.40		0.75		8.27	
Class (θ^2^/ψ^2^)	97.39		64.49		–	
K	–		–		185.37	
Grade 1	–		–		12.32	
Grade 2	–		–		10.17	
Grade 3	–		–		5.63	
Within (σ^2^)	165.12		145.92		–	
**MODEL FIT**						
Deviance	25,959		25,889		25,423	
AIC	25,987		25,917		25,479	
BIC	26,071		26,002		25,648	

*Lv: 3 level. Confidence intervals were obtained using profile likelihood method in xxM*.

Based on the average models as presented above and in Table 2, we have learned that the average EWPV for the control group at the beginning of kindergarten was 435.6 whereas the average EWPV score for the treatment group was slightly (but not significantly) lower (2.43 points lower). We have also learned that the average growth rate (or change) in EWPV was not a linear trend given that the regression coefficients of the two pieces were quite different from each other for both treatment and control groups (i.e., **13.75 piece1**_***tij***_ **+** **9.64 piece2**_***tij***_ for the control condition and **16.16 piece1**_***tij***_ **+** **11.24 piece2**_***tij***_ for the treatment condition). That is, we found a faster growth or improvement rate of EWPV within the kindergarten grade year and a slower growth rate of EWPV after kindergarten (i.e., from first to third grade) for both conditions, except that the students in the treatment condition, on average, showed greater improvement at the end of the kindergarten (16.16 points for the treatment condition vs. 13.75 points for the control condition) as well as at the end of first to third grade (11.24 points for the treatment condition vs. 9.64 points for the control condition). These differences in growth rates show the effectiveness of the Project ELLA enhanced materials and practice on improving the students' EWPV over time.

In general, researchers are more interested in the significance of the mean part (i.e., the regression coefficients) and pay less attention to the variance part of the model. Nevertheless, the variance part carries as much important information as the mean part (e.g., treatment effect is sometimes found in the variance part instead of the mean part of the model (Hedeker and Mermelstein, 2007), and the misspecification of the variance, in part, may lead to a biased estimation not only of the fixed effects (i.e., the regression coefficients) but also of the random effect variances (Sivo et al., 2005), which may further affect the significance tests of the regression coefficients (Kwok et al., 2007).

Given that we analyzed the data as a three-level, strictly hierarchical model, the corresponding variance estimates for the different levels are presented in Table 2 under the 3-Lv HLM column: σ^2^ = 165.12 (within-student-level variance with the identity structure assumption), τ_00_ = 137.36 (between-student-level intercept variance), τ_11_ = 2.13 (between-student-level piece1 variance), τ_22_ = .08 (between-student-level piece2 variance), and θ^2^ = 97.39 (***kindergarten*** classroom-level variance).

All these variances were statistically significant, which indicates a significant amount of variation within students across all the repeated measures and between students across all kindergarten classrooms. Consistent with many previous longitudinal studies using multilevel models, we found that the intercept variance (i.e., τ_00_ = 137.36) was, in general, substantial larger than the variances of the two growth pieces (i.e., τ_11_ = 2.13 and τ_22_ = 0.08).

There are a couple of limitations to this model. First, it only partially takes into account the classroom effect (i.e., only kindergarten), which may lead to biased estimation of both regression coefficients and the random effect variances. Moreover, only modeling the kindergarten effect restricts the possibility of modeling the other grade-level effects, such as the potential carryover effect from previous grade levels (e.g., first grade) to later EWPV score (e.g., measured at third grade).

## Model 2: the cross-classified random effect model (CCREM)

Another way to analyze this longitudinal data set is to apply the cross-classified random effect model (CCREM; Luo and Kwok, 2012). Although CCREM has been proposed for many years, this model is still not commonly applied in educational studies. In our study, we also provided useful information on how this model can be and was applied to a real, large scale randomized controlled longitudinal dataset. Unlike Model 1, which assumes a strictly hierarchical structure with repeated EWPV measures nested within students who further nested only within their kindergarten classrooms, the CCREM takes into account the classroom effects over time as a whole by creating a classroom crossed factor. In other words, instead of only considering the kindergarten classroom effect, the CCREM considers all (from kindergarten to third grade) classroom effects and assumes that at a given time point the only classroom effect present is the one at that particular time point. Additionally, classroom effects at different time points are interchangeable and, therefore, form one source of random effect variance. The setup of this model is similar to that of Model 1, as illustrated below.

**Level 1 (repeated-measure level)**

(5) $$\begin{array}{l} EWPV_{t(ij)}=\pi_{0(ij)}\;+\;\pi_{1(ij)}\;piece1_{t(ij)}\;+\;\pi_{2(ij)}\;piece2_{t(ij)}\\ \;+\;e_{t(ij)}, \end{array}$$

where EWPV is the target outcome variable for the t-th repeated measure from the i-th student of the j-th classroom and piece1 and piece2 are the time variables with the exact same coding scheme. The major difference between this model and Model 1 is the presentation and meaning of the subscript (specifically the “j” subscript). Unlike in Model 1 where the j subscript is only for a particular kindergarten classroom, the j subscript in Model 2 represents a particular classroom of any grade level (i.e., from kindergarten to third grade). That is, the students are no longer nested only within the kindergarten classrooms, as shown in Figure 1B. Instead, as shown in Figure 1A, the repeated measures are now nested within the i-th students and the j-th classroom whereas student and classroom are now crossed with each other. Hence the subscripts i and j in Equation (5) are now grouped in the parentheses (ij). For example, Student S1 in Figure 1A has three repeated measures (O_11_, O_12_ and O_13_), as does Student S2 (O_21_, O_22_ and O_23_). Students S1 and S2 are in different kindergarten classrooms (KC_1_ for S1 and KC_2_ for S2) but are in the same classroom in first grade (G1C_1_) and are assigned to different classrooms second grade (G2C_1_ for S1 and G2C_2_ for S2). Hence, the repeated measures (i.e., Os) are nested both within students (S1 and S2) and classrooms (KC_1_, KC_2_, G1C_1_, G2C_1_ and G2C_2_), whereas students and classrooms are crossed instead of nested.

Given that student and classrooms are crossed with each other, the level-2 model in CCREM includes both students and classrooms simultaneously as presented below:

**Level 2 (student and classroom level)**

(6) $$\begin{array}{l} \pi_{\text{0}(\text{ij})}=\gamma_{00}+\gamma_{01}treatment_{j}+u_{0i}+v_{0j}\\ \pi_{1(ij)}=\gamma_{10}+\gamma_{11}treatment_{j}+u_{1i}\\ \pi_{2(ij)}=\gamma_{20}+\gamma_{21}treatment_{j}+u_{2i}, \end{array}$$

where γ_**00**_, γ_**10**_, and γ_**20**_ are the average intercept, piece1 and piece2 coefficients across all classrooms, assuming the non-significant treatment effect. On the other hand, given that the randomization was at the classroom level, we included the dummy-coded treatment variable, treatment_*j*_, in the level-2 equations. Hence, if there is a significant treatment effect at intercept, γ_00_will be the intercept for the control condition whereas γ_00_ + γ_**01**_ will be the intercept for the treatment condition. Similarly, if there are significant treatment effects at both piece1 and piece2, the average piece1 coefficient will be γ_**10**_ for the control condition and γ_**10**_ + γ_**11**_ for the treatment condition; the same holds for the average piece2 coefficient, with γ_**20**_ for the control condition and γ_**20**_ + γ_**21**_ for the treatment condition.

By substituting Equation (6) back into Equation (5), we obtained the following overall ***average*** (or ***mean***) model, which is almost the same as Equation (4) under Model 1:

(7) $$\begin{array}{l} ÊWPV_{t(ij)}=\gamma_{00}+\gamma_{01}treatment_{j}+\gamma_{10}\;piece1_{t(ij)}\\ \;+\;\gamma_{20}\;piece2_{t(ij)}\;+\;\gamma_{11}treatment_{j}{\;}^{*}\;piece1_{t(ij)}\\ \;+\;\gamma_{21}treatment_{j}{\;}^{*}\;piece2_{t(ij)} \end{array}$$

The corresponding random effect variances are as follows:

V(***e***_***t***(***ij***)_) = σ^2^ (within-student-level variance with the identity structure assumption)

V(***u***_0***i***_) = τ_00_ (between-student-level intercept variance)

V(***u***_1***i***_) = τ_11_ (between-student-level piece1 variance)

V(***u***_2***i***_) = τ_22_ (between-student-level piece2 variance)

V(**v**_0j_) = ψ^2^ (between-classroom-level variance).

The major difference between this CCREM model and Model 1 is with regard to the random effect part; specifically, the classroom effect ***v***_0***j***_ with the corresponding variance equal to ψ^2^. Even though it seems like only a slight change in the combined equation (from ***v***_0***j***_ of the kindergarten random effects in Model 1 to ***v***_0***j***_ of all classroom random effects in Model 2), the actual implication and the parameter estimates of Model 2 can be very different from those of Model 1 due to the variance redistribution mechanism (Luo and Kwok, 2009). The corresponding output for this model may be found in Appendix A2. Below, we highlight these differences.

### Results of model 2

The results are presented in Table 2 in the Model 2 (CCREM) column. Instead of explaining each parameter estimate, we have highlighted the major differences between Models 1 and 2. First, the **treatment**_j_^*^ **piece2**_***t***(***ij***)_ interaction effect is no longer significant in Model 2 (γ_**21**_ =.64 with the 95% CI covered zero) compared with Model 1. This nonsignificant interaction effect indicates that the rate of change or improvement in the EWPV was the same for both treatment and control groups after kindergarten.

In addition to the regression coefficient, some of the estimates of the random effect variances were quite different between the two models: Model 2 had a larger intercept variance (τ_00_ = 157.04 compared with Model 1 τ_00_ = 137.36), a smaller classroom variance (ψ^2^ = 64.49 compared with Model 1 θ^2^ = 97.39), and a smaller within-student variance (σ^2^ = 145.92 compared with Model 1 σ^2^ = 165.12). These differences in the variance estimates between the two models are likely the result of the variance redistribution mechanism (Luo and Kwok, 2009). Although the number of parameters are the same in the two models, the meaning and setup (in terms of the design matrix) of the random effects, especially the classroom random effects, can result in quite different variance estimates which, in turn, can lead to different standard error estimates and tests of significance of the regression coefficients.

Regarding the limitation of this model, unlike Model 1 which only takes into account the kindergarten classroom effect, Model 2 is able to fully take the classroom effect into account. However, it does assume an acute classroom effect (i.e., it will not carry over in later grades). In other words, once a student changes grade (i.e., classroom), he/she will get a new classroom effect. The classroom effect at kindergarten is independent of the classroom effect at grade 1, for example. Also, all classroom effects regardless the grade (or time) have exactly the same variance given that they are treated as a whole or a single crossed factor, even though conceptually the classrooms at different grades/times may have different effects on the EWPV scores.

Ideally, we wanted to analyze this data set with four classroom crossed factors but, in reality, the specification for this model is not straightforward, especially when using the common MLM packages. Moreover, the model estimates only the variance for the classroom factors, not the other effects, such as the potential carryover effect from the previous classrooms on later EWPV scores.

## Model 3: xxM-UN1 piecewise latent growth

Whereas the nesting relationship holds in cross-sectional data, in longitudinal settings the relationship between students and classrooms is not pure. To make things more complicated, students' scores at a given time point, say second grade, are not only influenced by the classroom effect at second grade, but also potentially by the classroom effects at both kindergarten and first grade. Furthermore, the effect of the classroom may diminish, such that the impact of first grade may have a stronger effect on the second-grade scores than at third grade. Such a model would include five crossed random effects (i.e., one at the student level and four at the classroom level, including kindergarten, first-, second-, and third-grade random effects) and would need to allow the classroom effects to vary across time. None of the default models from the standard statistical packages can fully capture the key feature of this model.

**Figure 2:**
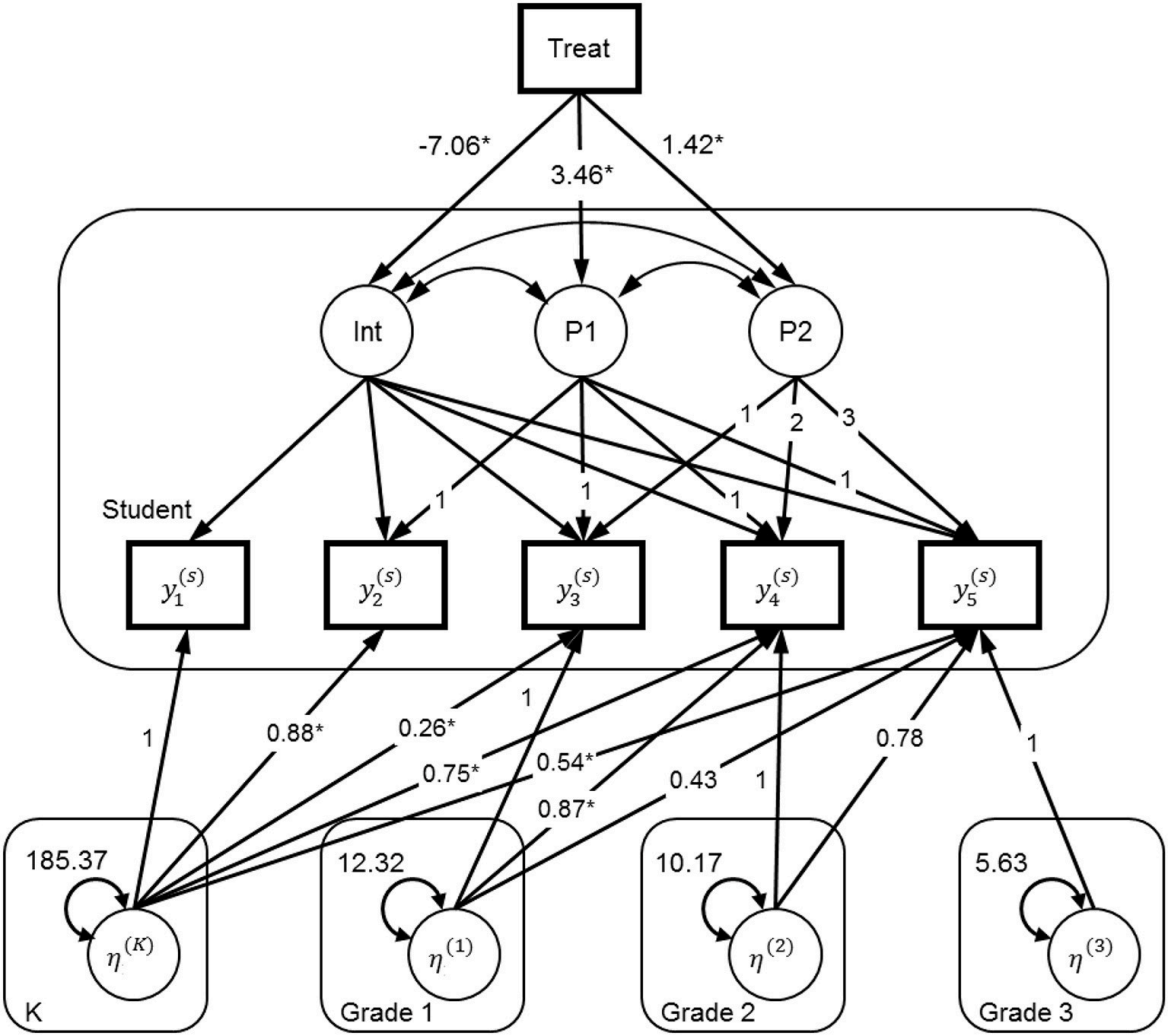
Path diagram for the model accommodating carryover classroom effects with five levels. y_1_, beginning of kindergarten; y_2_, end of kindergarten; y_3_, end of first grade; y_4_, end of second grade; y_5_, end of third grade. The rounded-corner boxes: Student, Student level; K, Kindergarten classroom level; Grade 1, Grade 1 classroom level; Grade 2, Grade 2 classroom level; Grade 3 classroom level.

Similar to Model 1, Model 3 (also see Figure 2) has also effectively captured the growth pattern and the treatment by pieces interaction effects after taking into account the data dependency. However, both Models 1 and 2 may not be the most optimal approach to analyze these data given some of the restricted assumptions. For example, they both assume that the residuals have a constant variance and are independent across time (i.e., an identity structure for the within-student variance-covariance structure). Moreover, they assume a constant classroom effect across time without any impact or carryover effect (from one grade level to the next).

The first limitation can be addressed by specifying a different residual covariance structure than the default one (see Kwok et al., 2007), which can be done in most multilevel software programs, such as HLM, SAS, and SPSS, as well as with the latent growth models under the SEM framework. The second limitation requires specification of multiple, crossed random effects to capture the potential non-constant classroom effects, which cannot be easily estimated in standard multilevel software^2^. Nevertheless, recent developments in the *n*-level SEM and the corresponding R package xxM (Mehta, 2013) have provided the potential to specify more complex multilevel models, including Model 3, as presented in Figure 2.

The model specification in xxM requires a combination of multilevel and SEM conventions. Due to its complexity, we only discuss the portions that are relevant to our model. First, it requires the longitudinal data to be in the wide rather than the long format (Kwok et al., 2008) in order to model complex residual covariance structures. This is identical to the latent growth modeling approach using SEM. Second, it requires a separate data set at each level, which is similar to the setup in HLM. In our model, we want to model five levels: the student level and four classrooms levels, including kindergarten (class-K), first grade (class-G1), second grade (class-G2), and third grade (class-G3). Third, it requires model specification at each level, and also for each pairwise combination of levels. For example, for a latent growth model with an additional classroom-level random effect, we have $y_{i}^{\left(1\right)}=\Lambda^{\left(1,1\right)}\eta_{i}^{\left(1\right)}+\Lambda^{\left(1,2\right)}\eta_{i}^{\left(2\right)}+\;\varepsilon_{i}^{\left(1\right)}$, where $y_{i}^{\left(1\right)}$ is the vector of the outcome scores of student *i* from Time 1 to Time 5, **Λ**^(1, 1)^ is a fixed pattern matrix for our piecewise growth model, $\eta_{i}^{\left(1\right)}$ is the vector of latent growth factor scores (i.e., intercept, piece1, and piece2) with mean **α**^(1)^ and variance-covariance matrix **Ψ**^(1, 1)^, and $\varepsilon_{i}^{\left(1\right)}$ is the student-level error terms. The superscripts ^(1)^ and ^(1, 1)^ denote a student-level model. At the classroom-level there is one latent variable $\eta_{i}^{\left(2\right)}$ denoting the random intercept, with mean **α**^(2)^ = 0 and variance **ψ**^(2)^, and with direct paths on ${\text{y}}_{i}^{\left(1\right)}$ through the between-class-K-student-level matrix **Λ**^(1, 2)^ = [1, 1, 1, 1, 1]^T^.

Because of the complexity associated with using xxM, we skip the model equations here to focus more on the conceptual formulation instead. The R code for fitting the model is presented in Appendix B.

### Student-level model

At the student level, we have a piecewise latent growth model for the five EWPV measurement occasions, which is equivalent to the piecewise growth model with random intercept and random coefficients for both piece1 and piece2, as opposed to lme4 (Bates et al., 2015), which requires the residual covariance structure to be a constant σ^2^ over time (i.e., an identity (ID) structure) as follows:

$$\sigma^{2}\left[\begin{array}{ccccc} 1 & 0 & 0 & 0 & 0\\ 0 & 1 & 0 & 0 & 0\\ 0 & 0 & 1 & 0 & 0\\ 0 & 0 & 0 & 1 & 0\\ 0 & 0 & 0 & 0 & 1 \end{array}\right].$$

In xxM, we can model many other kinds of structure, such as freely estimating the residual variances for different time points (i.e., the first-order unstructured [UN1] structure), as presented here in which the residual variances vary across time measures.

$$\left[\begin{array}{ccccc} \sigma_{A}^{2} & 0 & 0 & 0 & 0\\ 0 & \sigma_{B}^{2} & 0 & 0 & 0\\ 0 & 0 & \sigma_{C}^{2} & 0 & 0\\ 0 & 0 & 0 & \sigma_{D}^{2} & 0\\ 0 & 0 & 0 & 0 & \sigma_{E}^{2} \end{array}\right]$$

This seems to be a more realistic choice than the ID structure. The treatment condition that was assigned at kindergarten or as a class-K level variable predicts the intercept and the two piecewise growth factors (P1 and P2 in Figure 2). The corresponding path coefficients (of the paths/arrows from Treat to the growth latent factors in Figure 2) are conceptually equivalent to γ_**01**_ (**treatment**_***j***_), γ_11_(**treatment**_j_^*^ **piece1**_***t***(***ij***)_) and γ_21_(**treatment**_j_^*^ **piece2**_***t***(***ij***)_) in the previous two models.

### Four classroom-level models

At the class-K level, we have a random intercept factor η^(K)^ that accounts for the variance at all five time points due to clustering at kindergarten. We let the effect of such clustering differ across time points, which is achieved by allowing the direct paths (or factor loadings) from η^(K)^ to be different on the five measurement occasions. It is reasonable to expect that the effect will diminish across time, which means that the factor loadings should be decreasing. At the class-G1 level, we again have a random intercept factor η^(G1)^ that accounts for the clustering at first grade. Because classroom effect at first grade cannot affect prior performance (i.e., at kindergarten), the factor loadings from $\eta_{1}^{\left(\text{G}1\right)}$ to the first two measures are fixed at zero. Similar procedures are carried out for the remaining two random intercept factors, η^(G2)^ and η^(G3)^, as shown in Figure 2.

### Results of model 3

Given that the interpretation of the coefficients of the average or mean model is exactly the same as in the previous two models, we will focus more on the differences between Model 3 and the other two models. First, as shown in Table 2, all the fixed effects or regression coefficients were statistically significant with the 95% profile likelihood CI not covering zero. Specifically, when comparing the fixed effect estimates of Model 3 with those of the other two models, both coefficients of treatment (γ_**01**_ = −7.06) and Piece2 × Treatment (γ_**21**_ = 1.42) became significant.

**Figure 3:**
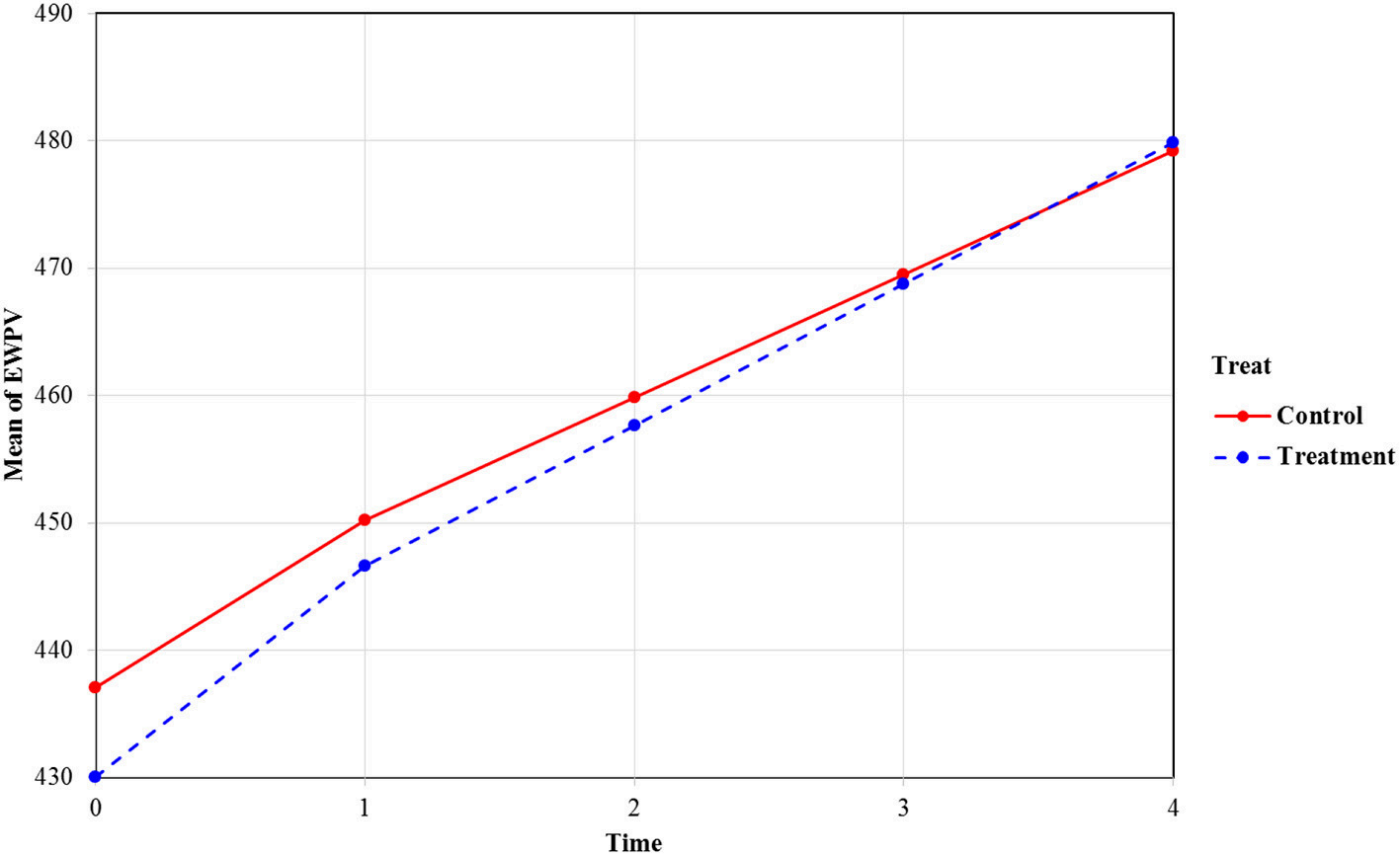
Mean trajectories of EWPV scores by the two treatment conditions. Value labels for the time axis: 0, beginning of kindergarten; 1, end of kindergarten; 2, end of first grade; 3, end of second grade; 4, end of third grade.

Figure 3 contains the estimated average models for both groups based on the estimates from the xxM-UN1 column in Table 2. As shown in Figure 3, the treatment group (the dashed line group) has lower EWPV scores at the beginning of kindergarten, and the growth (or improvement) rate of this group is faster than the control group at both pieces (i.e., the kindergarten piece and the first- to second-grade piece). The difference between the two groups on EWPV diminished as time passed, and by the end of second grade, the two lines crossed, which indicated no differences between the two groups. In other words, even though the treatment students started with significantly lower EWPV scores at the beginning of kindergarten, they caught up with their control group counterparts (by the end of second grade) and might even outperform them at the later time points. Notice also that the width of the CI is smaller for terms involving piece2 (compared with the corresponding terms involving piece1). This is likely a result of the decreasing classroom effect across time.

Another major difference between Model 3 and the previous two models is found in the variance part of the model: not only does Model 3 contain more random effects (i.e., four different classroom effects for the four different grades), but the sizes of the variance estimates (i.e., τ_00_, τ_11_, and τ_22_) are quite different from Models 1 and 2. As shown in Table 2, unlike the other two models with a single classroom variance, Model 3 contained four classroom variances for the four different grades, respectively.

A closer analysis of these classroom variances reveals that the kindergarten variance was the largest whereas the third-grade variance was the smallest. This trend and the substantial differences across grades may partly be the result of missing data—the missing data rate increased as time passed, and with fewer students at the later time points or grades, it is not surprising to see the diminished variance estimates. Other potential reasons may include the developmental process (i.e., students learn more when they grow older) and plausible treatment effect (e.g., students become more homogeneous/similar to each other when they respond to the treatment materials). Further investigation of this issue is needed.

For the same random effect variances (i.e., τ_00_, τ_11_, and τ_22_), Model 3 had substantially larger estimates than the other two models. This again may be the result of the variance redistribution mechanism (Luo and Kwok, 2009) due to the additional classroom variances. Given that the standard errors (SEs) of the fixed effect estimates (or regression coefficients) are a function of the random effect variances, the additional significant coefficients (i.e., γ_**01**_ & γ_**21**_) in Model 3 are likely the results of these different variance estimates, which can directly affect the tests of significance of these coefficients.

In addition to the fixed and random effect estimates commonly found in the traditional multilevel models and presented in Table 2, we further examined the potential carryover effect using xxM due to its flexibility of specifying more complex multilevel models. As shown in Figure 2, the direct paths (arrows) from each classroom factor to the individual time measures can be viewed as examining the carryover effect; that is, the effect from the previous grade classroom to the current and later time EWPV scores. For model identification, we constrained the direct path of the current time measure to 1.0 (e.g., fixing the kindergarten effect to Y1 [K-begin] to 1.0, while freely estimating other paths). As shown in Figure 2, the freely estimated direct paths from kindergarten to all the time measures (K-end, 1st-end, 2nd-end, and 3rd-end) were significant, with the largest effect at the immediate post measure (i.e., the end of kindergarten EWPV score) followed by weaker effects at later time measures.

We found a similar pattern for the first-grade factor (i.e., larger direct path coefficient to the immediate post measure followed by smaller coefficient to later time measures), even though the direct path coefficients were not all significant, possibly as a result of the smaller sample sizes at this grade and the later grade levels. Similar non-significant direct effects were also found for the second-grade factor.

These significant and non-significant carryover effects at different grade levels had some important and practical implications. For example, the many significant carryover effects from kindergarten may reflect the importance of the timing (i.e., the start of the intervention) and the potential longitudinal effect of the intervention. In other words, we may not see the same treatment effect if the intervention starts at another grade level as opposed to the beginning of kindergarten. Moreover, the significant paths from kindergarten to later-grade EWPV scores may reveal the importance of the kindergarten classroom experience, which may relate to ELL students' reading performance in the later grades, and further examination of this will be needed.

We compared the three models by using information criteria; namely, the Akaike information criterion (AIC) and the Bayesian information criterion (BIC). Certain guidelines apply to interpreting the absolute difference of the information criteria (i.e., ΔIC) between two competing models. For example, Burnham and Anderson (1998) suggested that when ΔAIC between two compared models is larger than 4, we can establish that the model with smaller AIC is better than the other model with larger AIC. Likewise, Raftery (1996) pointed out that the ΔBIC between two competing models should be at least 2 to indicate a real difference. Based on these guidelines, we found that Model 3 fit the data the best given the smallest AIC and BIC values across all three models.

## Discussion

In this study, we first described the complexity of the educational data, especially in longitudinal settings, which can result in data with a non-strictly hierarchical but more complex multilevel structure. With the use of the ELLA data, we demonstrated the importance of capturing the complex data structure by examining three different models with different random effect specification.

As stated, researchers are generally interested in the overall average model (or the mean part of the model containing the regression coefficients), but they fail to pay close attention to the variance part of the model. Yet, the variance part also carries important information, such as the implication of the developmental process. We have discussed and shown the importance of carefully specifying the random part of the model, which could affect estimation of the random effect variances and further affect estimates of the standard errors of the regression coefficients and the corresponding significance tests of these coefficients. For example, we found that both Models 1 and 3 had significant treatment by pieces interaction effects whereas Model 2 only had significant treatment by piece1 interaction effect and only contained some but not all significant coefficients. This finding provides evidence that only partially addressing the complex data structure may result in lower statistical power and loss of some important findings such as the treatment by growth piece (i.e. piece2 covering changes from the end of first to end of third grade) interaction effect.

Another advantage of modeling the classroom effect by grade levels separately (i.e., Model 3) instead of as a whole (e.g., Model 2 using CCREM) is that it allows researchers to investigate interesting phenomena that cannot be captured by the mean part of the model. For example, the decreasing classroom or grade variances over time may reflect the important developmental process. For example, the high heterogeneity (or variation) among students at the beginning of kindergarten may be the result of the diverse backgrounds and experiences the students have before they entered formal schooling. Once they are exposed to the formal grade-school curriculum in addition to their natural cognitive development, the variation among the students may become smaller, which in turn, may lead to a reduction in grade-level variances over time.

This is a plausible explanation, but further systematic investigation on the change in the variances is needed to validate this interpretation. Again, researchers should not only focus on the mean part of the model (i.e., the significance of the regression coefficients), but also, they should examine different random effect structure, which may provide different perspectives and even lead to new research questions for the target phenomena.

Moreover, we have shown how to incorporate the carryover effect in the model via the xxM program. The pattern of the carryover effect has shed light on some important and practical design issues, such as the timing of the study and the potential longitudinal impact of the intervention. For example, the only significant carryover effects from the kindergarten factor to the later time measures may suggest the importance of starting this type of intervention at kindergarten (rather than at other/later grade level). In fact, such carryover impact was also supported by empirical evidence on Project ELLA students' subsequent learning as they matriculated to grade 5 (e.g., Tong et al., 2014).

Despite the important results presented here, there are a few limitations to the study. First, even though xxM is a very powerful software for very complex multilevel data, its lack of model-fit indices (e.g., RMSEA and CFI) restricts researchers to evaluate their models only based on the deviance statistic and the information criteria. Similarly, an appropriate standardized effect size measure for this type of complex data structure has not yet been developed. Another major limitation is that we only used real data for the demonstration. Thus, the actual impact of various factors such as the magnitude of the data dependency (or intra-class correlation) and the missing data rate over time can only be further examined by thoughtfully planned simulation studies. Moreover, the carry-over effects found in Model 3 (also see Figure 2) are in arbitrary metric, and researchers need to be cautious when interpreting these findings. Besides xxM, a similar type of model (Model 3) may possibly specify and analyze with non-SEM Bayesian based programs such as STAN (Carpenter et al., 2017). Further investigation on whether and how effective this alternative approach on fitting the same type of carry-over effect model to similar real, large scale randomized controlled longitudinal data will be needed.

When analyzing complex longitudinal data, especially those from different educational settings, researchers generally focus only on the mean part (i.e., the regression coefficients) while ignoring the equally important random part (i.e., the random effect variances) of the model. Throughout this paper, we have addressed the importance of adequately taking the complex data structure into account by carefully specifying the random part of the model—not only can it affect the variance estimates, the standard errors, and the tests of significance of the regression coefficients, it can also offer additional information such as the potential developmental process and the carryover effect. We used xxM, which allowed us to estimate different grade level variances (i.e., from kindergarten to third grade, separately) and the potential carryover effect from each grade factor to the later time measures of the EWPV scores. In closing, we encourage researchers to look beyond the mean part of the model (i.e., the regression coefficients) and explore the variance part of the model that may lead them to different perspectives or even new information of the phenomena they are studying.

## Author contributions

O-MK and ML are the lead authors who wrote most of the manuscript and conducted all the analyses. Other coauthors contributed on providing the data and related information FT, RL-A, and BI) and offering constructive feedback to the manuscript FT, RL-A, BI, MY, and Y-CY).

### Conflict of interest statement

The authors declare that the research was conducted in the absence of any commercial or financial relationships that could be construed as a potential conflict of interest.

## Footnotes

^1^The reason of including only one random effect (i.e., ***v***_0***j***_) at the classroom level in equation (3) is to have a simpler model (in terms of the number of random effects) to avoid the potential convergence issue due to the large number of variance and covariance estimates of the random effects. Additionally, according to our experience, the variance estimates of the higher level non-intercept random effects are generally very small and non-significant and trying to estimate these tiny (and possibly non-significant) random effect variances will likely lead to non-converged result.

^2^To our best knowledge, across all the commercial SEM related software, only M*plus* and Stata (the “gsem” routine) can handle cross-classified data with limited number of crossed factors (e.g., Mplus can only handle two crossed factors).

## References

[B1] Bates D. Mächler M. Bolker B. Walker S. (2015). Fitting linear mixed-effects models using lme4. J. Stat. Softw. 67, 1–48. 10.18637/jss.v067.i01

[B2] Bollen K. A. Curran P. J. (2006). Latent Curve Models: A Structural Equation Modeling Perspective. Hoboken, NJ: Wiley.

[B3] Bryk A. S. Raudenbush S. W. (1992). Hierarchical Linear Models: Applications and Data Analysis Methods. Newbury Park, CA: Sage.

[B4] Burnham K. P. Anderson D. R. (1998). Model Selection and Inference: A Practical Information-Theoretic Approach. New York, NY: Springer-Verlag.

[B5] Carpenter B. Gelman A. Hoffman M. D. Lee D. Goodrich B. Betancourt M. (2017). Stan: A probabilistic programming language. J. Stat. Softw. 76, 1–32. 10.18637/jss.v076.i01 PMC9788645 36568334

[B6] Curran P. J. (2003). Have multilevel models been structural equation models all along? Multiv. Behav. Res. 38, 529–569. 10.1207/s15327906mbr3804_5 26777445

[B7] Duncan T. E. Duncan S. C. Strycker L. A. (2006). An Introduction to Latent Variable Growth Curve Modeling: Concepts, Issues, and Applications, 2nd Edn. Mahwah, NJ: Lawrence Erlbaum.

[B8] Hedeker D. Mermelstein R. J. (2007). Mixed-effects regression models with heterogeneous variance: analyzing ecological momentary assessment data of smoking, in Modeling Contextual Effects in Longitudinal Studies, eds Little T. D. Bovaird J. A. Card N. A. (Mahwah, NJ: Lawrence Erlbaum), 183–206.

[B9] Kwok O. Luo W. West S. G. (2010). Using modification indexes to detect turning points in longitudinal data: A Monte Carlo study. Struc. Equat. Model. 17, 216–240. 10.1080/10705511003659359

[B10] Kwok O. Underhill A. T. Berry J. W. Luo W. Elliott T. R. Yoon M. (2008). Analyzing longitudinal data with multilevel models: an example with individuals living with lower extremity intra-articular fractures. Rehabil. Psychol. 53, 370–386. 10.1037/a0012765 19649151 PMC2613314

[B11] Kwok O. West S. G. Green S. B. (2007). The impact of misspecifying the within-subject covariance structure in multilevel longitudinal multilevel models: a Monte Carlo study. Multiv. Behav. Res. 42, 557–592. 10.1080/00273170701540537

[B12] Lara-Alecio R. (2003). English Language and Literacy Acquisition (Project ELLA). Washington, DC: U.S. Department of Education Available online at: http://epsy.tamu.edu/sites/epsy.tamu.edu/files/Lara-Alecio-Project%20ELLA.pdf

[B13] Luo W. Kwok O. (2009). The impacts of ignoring a crossed factor in analyzing cross-classified data. Multivariate Behav. Res. 44, 182–212. 10.1080/00273170902794214 26754266

[B14] Luo W. Kwok O. (2012). The consequences of ignoring individuals' mobility in multilevel growth models: A Monte Carlo study. J. Educ. Behav. Stat. 37, 31–56 10.3102/1076998610394366

[B15] Mehta P. (2013). xxM: Structural Equation Modeling for Dependent Data (R package version 0.6.0) [Computer program]. Available online at: http://xxm.times.uh.edu/

[B16] Meyers J. Beretvas S. N. (2006). The impact of inappropriate modeling of cross-classified data structures. Multiv. Behav. Res. 41, 473–497. 10.1207/s15327906mbr4104_3 26794915

[B17] Preacher K. Wichman A. MacCallum R. Briggs N. (2008). Latent Growth Curve Modeling. Thousand Oaks, CA: Sage.

[B18] Raftery A. E. (1996). Bayesian model selection in social research. Sociol. Methodol. 25, 111–163. 10.2307/271063

[B19] Sayer A. G. Willett J. B. (1998). A cross-domain model for growth in adolescent alcohol expectancies. Multiv. Behav. Res. 33, 509–543. 10.1207/s15327906mbr3304_4 26753827

[B20] Sivo S. Fan X. Witta L. (2005). The biasing effects of unmodeled ARMA time series processes on latent growth curve model estimates. Struct. Equ. Model. 12, 215–231. 10.1207/s15328007sem1202_2

[B21] Snijders T. A. B. Bosker R. (1999). Multilevel Analysis: An Introduction to Basic and Advanced Multilevel Modeling. Newbury Park, CA: Sage.

[B22] Texas Education Code (1995). 74th Leg., Ch. 260, Section 29.056, § 1

[B23] Tong F. Irby B. J. Lara-Alecio R. Koch J. (2014). A longitudinal study of integrating literacy and science for fifth grade Hispanic current and former English language learners: from learning to read to reading to learn. J. Educ. Res. 107, 410–426. 10.1080/00220671.2013.833072

