# Supplementary material for: Analyzing Complex Longitudinal Data in Educational Research: A Demonstration With Project English Language and Literacy Acquisition (ELLA) Data Using xxM
Source: Front Psychol. 2018 Jun 5;9:790. doi: 10.3389/fpsyg.2018.00790 (PMC5996051; doi:10.3389/fpsyg.2018.00790)
Supplement: Supplementary file 1 [file Data_Sheet_1.docx]

**Appendix A1: xxM Output for Model 1 (3-Lv HLM)**

$fit

$fit$deviance

[1] 25958.53

$fit$nParameters

[1] 14

$fit$nObservations

[1] 3122

$fit$aic

[1] 25986.53

$fit$bic

[1] 26071.18

$estimates

child parent to from label estimate low high

1 SMUID1 SMUID1 EWPV1 EWPV1 theta 165.12 155.71 175.25

3 SMUID1 SMUID1 EWPV2 EWPV2 theta 165.12 155.71 175.25

6 SMUID1 SMUID1 EWPV3 EWPV3 theta 165.12 155.71 175.25

10 SMUID1 SMUID1 EWPV4 EWPV4 theta 165.12 155.71 175.25

15 SMUID1 SMUID1 EWPV5 EWPV5 theta 165.12 155.71 175.25

16 SMUID1 SMUID1 int int evar_int 137.36 107.72 171.86

17 SMUID1 SMUID1 int p1 ecov_int_p1 -17.10 0.00 0.00

18 SMUID1 SMUID1 p1 p1 evar_p1 2.13 0.00 13.97

19 SMUID1 SMUID1 int p2 ecov_int_p2 -3.22 0.00 0.00

20 SMUID1 SMUID1 p1 p2 ecov_p1_p2 0.40 0.00 0.00

21 SMUID1 SMUID1 p2 p2 evar_p2 0.08 0.00 1.47

22 SMUID1 SMUID1 int One mean_int 435.60 432.91 438.28

23 SMUID1 SMUID1 p1 One mean_p1 13.75 11.96 15.54

24 SMUID1 SMUID1 p2 One mean_p2 9.64 8.95 10.34

45 SMUID1 classK int treat treat_int -2.43 -7.41 2.61

46 SMUID1 classK p1 treat treat_p1 2.41 0.01 4.80

47 SMUID1 classK p2 treat treat_p2 1.60 0.59 2.62

48 classK classK eta_K eta_K var_K 97.39 63.92 152.75

**Appendix A2: xxM Output for Model 2 (CCREM)**

$fit

$fit$deviance

[1] 25889.31

$fit$nParameters

[1] 14

$fit$nObservations

[1] 3122

$fit$aic

[1] 25917.31

$fit$bic

[1] 26001.95

$estimates

child parent to from label estimate low high

1 SMUID1 SMUID1 EWPV1 EWPV1 theta 145.92 137.35 155.20

3 SMUID1 SMUID1 EWPV2 EWPV2 theta 145.92 137.35 155.20

6 SMUID1 SMUID1 EWPV3 EWPV3 theta 145.92 137.35 155.20

10 SMUID1 SMUID1 EWPV4 EWPV4 theta 145.92 137.35 155.20

15 SMUID1 SMUID1 EWPV5 EWPV5 theta 145.92 137.35 155.20

16 SMUID1 SMUID1 int int evar_int 157.04 127.46 191.13

17 SMUID1 SMUID1 int p1 ecov_int_p1 -23.50 0.00 0.00

18 SMUID1 SMUID1 p1 p1 evar_p1 3.52 0.29 11.78

19 SMUID1 SMUID1 int p2 ecov_int_p2 -5.02 0.00 0.00

20 SMUID1 SMUID1 p1 p2 ecov_p1_p2 0.75 0.00 0.00

21 SMUID1 SMUID1 p2 p2 evar_p2 0.16 0.00 1.24

22 SMUID1 SMUID1 int One mean_int 436.99 434.31 439.67

23 SMUID1 SMUID1 p1 One mean_p1 13.15 11.36 14.94

24 SMUID1 SMUID1 p2 One mean_p2 9.47 8.23 10.71

45 SMUID1 classK int treat treat_int -3.12 -7.20 1.03

46 SMUID1 classK p1 treat treat_p1 3.42 1.04 5.81

47 SMUID1 classK p2 treat treat_p2 0.59 -1.36 2.53

48 classK classK eta_K eta_K var_class 64.49 47.63 86.85

54 class1 class1 eta_G1 eta_G1 var_class 64.49 47.63 86.85

60 class2 class2 eta_G2 eta_G2 var_class 64.49 47.63 86.85

66 class3 class3 eta_G3 eta_G3 var_class 64.49 47.63 86.85

**Appendix A3: xxM Output for Model 3 (xxM-UN1)**

$fit

$fit$deviance

[1] 25418.12

$fit$nParameters

[1] 28

$fit$nObservations

[1] 3122

$fit$aic

[1] 25474.12

$fit$bic

[1] 25643.41

$estimates

child parent to from label estimate low high

1 SMUID1 SMUID1 EWPV1 EWPV1 theta1 86.05 0.00 145.24

3 SMUID1 SMUID1 EWPV2 EWPV2 theta2 97.81 72.09 -99999.99

6 SMUID1 SMUID1 EWPV3 EWPV3 theta3 319.30 282.59 361.88

10 SMUID1 SMUID1 EWPV4 EWPV4 theta4 52.86 42.51 65.10

15 SMUID1 SMUID1 EWPV5 EWPV5 theta5 38.51 23.61 -99999.99

16 SMUID1 SMUID1 int int evar_int 193.44 127.58 -99999.99

17 SMUID1 SMUID1 int p1 ecov_int_p1 -39.80 -147.13 16.83

18 SMUID1 SMUID1 p1 p1 evar_p1 29.37 0.00 -99999.99

19 SMUID1 SMUID1 int p2 ecov_int_p2 -13.56 -21.48 -5.91

20 SMUID1 SMUID1 p1 p2 ecov_p1_p2 8.27 -1.92 -99999.99

21 SMUID1 SMUID1 p2 p2 evar_p2 3.66 0.65 8.17

22 SMUID1 SMUID1 int One mean_int 437.07 434.16 440.00

23 SMUID1 SMUID1 p1 One mean_p1 13.12 11.51 14.72

24 SMUID1 SMUID1 p2 One mean_p2 9.66 8.90 10.44

41 SMUID1 classK EWPV2 eta_K K_to_t2 0.88 0.81 0.97

42 SMUID1 classK EWPV3 eta_K K_to_t3 0.26 0.11 0.41

43 SMUID1 classK EWPV4 eta_K K_to_t4 0.75 0.62 0.87

44 SMUID1 classK EWPV5 eta_K K_to_t5 0.54 0.42 0.67

45 SMUID1 classK int treat treat_int -7.06 -11.96 -1.94

46 SMUID1 classK p1 treat treat_p1 3.46 1.32 5.63

47 SMUID1 classK p2 treat treat_p2 1.42 0.23 2.57

48 classK classK eta_K eta_K var_K 185.37 122.91 288.78

52 SMUID1 class1 EWPV4 eta_G1 G1_to_t4 0.87 0.20 4.26

53 SMUID1 class1 EWPV5 eta_G1 G1_to_t5 0.43 -0.22 2.37

54 class1 class1 eta_G1 eta_G1 var_G1 12.32 0.66 35.40

59 SMUID1 class2 EWPV5 eta_G2 G2_to_t5 0.78 -191.30 193.14

60 class2 class2 eta_G2 eta_G2 var_G2 10.17 0.00 26.53

66 class3 class3 eta_G3 eta_G3 var_G3 5.63 0.65 15.35

**Appendix B: Annotated xxM Input Syntax (for Model 3: xxM-UN1)**

library(xxm) # load the `xxm` package

# see http://xxm.times.uh.edu/download-2/ for installation instruction

head(dat1w) # summary of the data

# Variable description:

# Outcomes: EWPV1, EWPV2, EWPV3, EWPV4, EWPV5

# Student identifier: SMUID1

# Classroom ID: classK, class1, class2, class3

# Intervention indicator: treat (0 = conventional, 1 = experimental)

# Prepare the Data --------------------------------------------------------

# Data for the student-level:

ewpv.S <- dat1w[, c("SMUID1", "classK", "class1", "class2", "class3",

"treat", paste0("EWPV", 1:5))]

# Data for kindergarten-level:

ewpv.K <- aggregate(treat ~ classK, data = dat1w, FUN = mean)

# Data for grade-1-level:

ewpv.G1 <- aggregate(treat ~ class1, data = dat1w, FUN = mean)

# Data for grade-2-level:

ewpv.G2 <- aggregate(treat ~ class2, data = dat1w, FUN = mean)

# Data for grade-3-level:

ewpv.G3 <- aggregate(treat ~ class3, data = dat1w, FUN = mean)

# Initialize the Model and Submodels --------------------------------------

# Overall model (5 levels)

m_carry <- xxmModel(levels = c("SMUID1",

"classK", "class1", "class2", "class3"))

# Student-level submodel (4 parents,

# 5 endogenous variables,

# 3 latent variables)

m_carry <- xxmSubmodel(model = m_carry,

level = "SMUID1",

parents = c("classK", "class1", "class2", "class3"),

ys = c("EWPV1", "EWPV2", "EWPV3", "EWPV4", "EWPV5"),

xs = ,

etas = c("int", "p1", "p2"),

data = ewpv.S)

# Kindergarten-level submodel (no parents,

# 1 exogenous variable,

# 1 latent variable)

m_carry <- xxmSubmodel(model = m_carry,

level = "classK",

parents = ,

ys = ,

xs = "treat",

etas = "eta_K",

data = ewpv.K)

# Grade-1 submodel (no parents,

# 1 exogenous variable,

# 1 latent variable)

m_carry <- xxmSubmodel(model = m_carry,

level = "class1",

parents = ,

ys = ,

xs = ,

etas = "eta_G1",

data = ewpv.G1)

# Grade-2 submodel (no parents,

# 1 exogenous variable,

# 1 latent variable)

m_carry <- xxmSubmodel(model = m_carry,

level = "class2",

parents = ,

ys = ,

xs = ,

etas = "eta_G2",

data = ewpv.G2)

# Grade-3 submodel (no parents,

# 1 exogenous variable,

# 1 latent variable)

m_carry <- xxmSubmodel(model = m_carry,

level = "class3",

parents = ,

ys = ,

xs = ,

etas = "eta_G3",

data = ewpv.G3)

# Define the Within-Level Submodels ---------------------------------------

# Notes: here are how we name the matrices:

# "th" (Theta) = residual covariance structure,

# "ps" (Psi) = latent factor covariance matrix,

# "al" (alpha) = latent factor means (or intercepts),

# "ly" (Lambda-Y) = factor loading matrix,

# "ga" (Gamma) = path coefficient matrices

#

# and each matrix is defined by

# "_pat" = indicating whether each element is fixed (= 0) or free (= 1)

# "_val" = containing the starting (if free)/assigned (if fixed) values

# "_lab" = optional labels given to the free parameters

# Student-level

# Theta (covariance matrix with an UN(1) structure):

th_pat <- diag(5) # 5x5 diagonal matrix

th_val <- diag(100, 5)

th_lab <- diag(0, 5) # initialize an empty matrix

diag(th_lab) <- paste0("theta", 1:5) # give labels to the diagonal

# Psi (latent growth factor covariances):

ps_pat <- matrix(1, nrow = 3, ncol = 3)

ps_val <- diag(c(124, 20, 20))

ps_lab <- diag(0, 3)

diag(ps_lab) <- paste0("evar_", c("int", "p1", "p2"))

ps_lab[lower.tri(ps_lab)] <- c("ecov_int_p1", "ecov_int_p2", "ecov_p1_p2")

ps_lab[upper.tri(ps_lab)] <- c("ecov_int_p1", "ecov_int_p2", "ecov_p1_p2")

# alpha (growth factor means):

al_pat <- matrix(1, nrow = 3, ncol = 1)

al_val <- matrix(c(437, 13, 10), ncol = 1)

al_lab <- matrix(c("mean_int", "mean_p1", "mean_p2"), nrow = 3)

# Lambda-Y (pattern coefficients from growth factors to the

# observed variables):

ly_pat <- matrix(0, nrow = 5, ncol = 3)

ly_val <- matrix(c(rep(1, 5), 0, rep(1, 4), 0, 0, 1:3), nrow = 5)

# Now, assemble the above to the within-student-level matrix

m_carry <- xxmWithinMatrix(m_carry, "SMUID1", "theta", th_pat, th_val, th_lab)

m_carry <- xxmWithinMatrix(m_carry, "SMUID1", "psi", ps_pat, ps_val, ps_lab)

m_carry <- xxmWithinMatrix(m_carry, "SMUID1", "alpha", al_pat, al_val, al_lab)

m_carry <- xxmWithinMatrix(m_carry, "SMUID1", "lambda", ly_pat, ly_val)

# Kindergarten level

# Psi (random effect variance):

K_ps_pat <- matrix(1)

K_ps_val <- matrix(100)

K_ps_lab <- matrix("var_K")

# Now, assemble the above to the within-kindergarten-level matrix

m_carry <- xxmWithinMatrix(m_carry, "classK", "psi",

K_ps_pat, K_ps_val, K_ps_lab)

# Grade 1 level

G1_ps_pat <- matrix(1)

G1_ps_val <- matrix(22)

G1_ps_lab <- matrix("var_G1")

# Now, assemble the above to the within-grade-1-level matrix

m_carry <- xxmWithinMatrix(m_carry, "class1", "psi",

G1_ps_pat, G1_ps_val, G1_ps_lab)

# Grade 2 level

G2_ps_pat <- matrix(1)

G2_ps_val <- matrix(36)

G2_ps_lab <- matrix("var_G2")

# Now, assemble the above to the within-grade-2-level matrix

m_carry <- xxmWithinMatrix(m_carry, "class2", "psi",

G2_ps_pat, G2_ps_val, G2_ps_lab)

# Grade 3 level

G3_ps_pat <- matrix(1)

G3_ps_val <- matrix(15)

G3_ps_lab <- matrix("var_G3")

# Now, assemble the above to the within-grade-3-level matrix

m_carry <- xxmWithinMatrix(m_carry, "class3", "psi",

G3_ps_pat, G3_ps_val, G3_ps_lab)

# Define the Between-Level Submodels --------------------------------------

# Kindergarten -> Student

# Lambda-Y (fixed the first to 1 for identification):

K_ly_pat <- matrix(c(0, rep(1, 4)), ncol = 1)

K_ly_val <- matrix(c(1, 1, .6, .4, .2), nrow = 5)

K_ly_lab <- matrix(c("K_to_t1", "K_to_t2", "K_to_t3", "K_to_t4", "K_to_t5"))

# Gamma (intervention effects)

K_ga_pat <- matrix(c(1, 1, 1), nrow = 3, ncol = 1)

K_ga_val <- matrix(c(-3, 3, 2), nrow = 3)

K_ga_lab <- matrix(c("treat_int", "treat_p1", "treat_p2"), nrow = 3)

# Now, assemble the above to the K-student between-level matrix

m_carry <- xxmBetweenMatrix(m_carry, parent = "classK", child = "SMUID1",

type = "lambda", pattern = K_ly_pat,

value = K_ly_val, label = K_ly_lab)

m_carry <- xxmBetweenMatrix(m_carry, parent = "classK", child = "SMUID1",

type = "gamma", pattern = K_ga_pat,

value = K_ga_pat, label = K_ga_lab)

# G1 -> Student

# Lambda-Y (influence starts at T3):

G1_ly_pat <- matrix(c(rep(0, 3), rep(1, 2)), ncol = 1)

G1_ly_val <- matrix(c(0, 0, 1, .8, .6), nrow = 5)

G1_ly_lab <- matrix(c("G1_to_t1", "G1_to_t2", "G1_to_t3",

"G1_to_t4", "G1_to_t5"))

# Now, assemble the above to the G1-student between-level matrix

m_carry <- xxmBetweenMatrix(m_carry, parent = "class1", child = "SMUID1",

type = "lambda", pattern = G1_ly_pat,

value = G1_ly_val, label = G1_ly_lab)

# G2 -> Student

# Lambda-Y (influence starts at T4):

G2_ly_pat <- matrix(c(rep(0, 4), 1), ncol = 1)

G2_ly_val <- matrix(c(0, 0, 0, 1, .5), nrow = 5)

G2_ly_lab <- matrix(c("G2_to_t1", "G2_to_t2", "G2_to_t3",

"G2_to_t4", "G2_to_t5"))

# Now, assemble the above to the G2-student between-level matrix

m_carry <- xxmBetweenMatrix(m_carry, parent = "class2", child = "SMUID1",

type = "lambda", pattern = G2_ly_pat,

value = G2_ly_val, label = G2_ly_lab)

# G3 -> Student

# Lambda-Y (influence starts at T4):

G3_ly_pat <- matrix(0, nrow = 5, ncol = 1)

G3_ly_val <- matrix(c(rep(0, 4), 1), nrow = 5)

# Now, assemble the above to the G3-student between-level matrix

m_carry <- xxmBetweenMatrix(m_carry, parent = "class3", child = "SMUID1",

type = "lambda", pattern = G3_ly_pat,

value = G3_ly_val)

# Run the Model With xxM --------------------------------------------------

m_carry <- xxmRun(m_carry) # get the estimates with maximum likelihood

m_carry <- xxmCI(m_carry) # get Profile Likelihood CI

# xxmSummary(m_carry) # print model summary

m_carry_summary <- xxmSummary(m_carry)

m_carry_summary$estimates[6:8] <- round(m_carry_summary$estimates[6:8], 2)

sink('xxm_pw_un1_fixed.txt')

m_carry_summary

sink()

xxmFree(m_carry)
